# Spatial mapping of CoQ10 repletion by BPM31510 in a genetic mouse model (*Coq4*^*F147C*^) of coenzyme Q deficiency

**DOI:** 10.1016/j.jlr.2026.100987

**Published:** 2026-01-29

**Authors:** Eliana Barriocanal-Casado, Sylwia A. Stopka, Alba Pesini, Juan J. Aristizabal-Henao, Srada Karmacharya, Devon Van Cura, Ryan Zhang, Kelsey R. Nickerson, Kashni Grover, Oksana Zavidij, Sarah R. Wessel, Niven R. Narain, Vijay Modur, Stephane Gesta, Michael A. Kiebish, Catarina M. Quinzii

**Affiliations:** 1 Department of Neurology, Columbia University Irving Medical Center, New York, NY, USA; 2 BPGbio, Waltham, MA, USA; 3 Department of Molecular Biology, Dermatology and Cutaneous Surgery, University of Miami Miller School of Medicine, Miami, FL, USA

**Keywords:** CoQ10 deficiency, mitochondrial disease, MS, quinomics, MALDI, MSI

## Abstract

Primary coenzyme Q10 (CoQ10) deficiency is a rare mitochondrial disorder caused by mutations in genes involved in CoQ biosynthesis (e.g., *COQ4*) that result in impaired mitochondrial respiration, oxidative stress, and dysfunction across multiple organ systems because of decreased mitochondrial levels of CoQ10. Although oral CoQ10 supplementation has been examined for standard of care, poor absorption and inadequate tissue and intracellular distribution have resulted in a lack of clinically significant efficacy. BPM31510 is a lipid nanoparticle containing oxidized CoQ10 designed to improve bioavailability and targeted uptake into the mitochondria. In the current study, we assessed the efficacy of BPM31510 to increase CoQ levels in *Coq4*^*F147C*^ mice, a novel genetic knock-in model of primary CoQ deficiency. Coenzyme Q9, the main form of CoQ in mice, and CoQ10 were significantly decreased in the brain, kidney, heart, and muscle of *Coq4*^*F147C*^ mice compared with *Coq4*^*+/+*^ mice. BPM31510 treatment significantly increased oxidized CoQ10 levels across all tissues, mediated by the nanoliposome biodistribution of oxidized CoQ10 in BPM31510. MALDI-MS imaging demonstrated regional and spatial restoration of CoQ10 within the brain, including the cerebellum, myocardium, and renal cortex of *Coq4*^*F147C*^ mice. These results demonstrate that BPM31510 successfully concentrates pharmacologically active CoQ10 in target tissues that are not reachable with oral therapy in a genetic model of primary CoQ deficiency. We enabled the visualization of suborgan CoQ10 localization to specifically demonstrate CoQ10 restoration. This study establishes proof of concept for spatial quinomics, a new methodology that combines spatial metabolomics with quinomics to evaluate next-generation CoQ10-based therapeutics for mitochondrial disorders.

Coenzyme Q (CoQ), also known as ubiquinone (for CoQ10), is a lipophilic molecule that is required for mitochondrial metabolism and homeostasis (1), including mitochondrial respiration. CoQ is composed of a benzoquinone ring and an isoprenoid chain of variable lengths. The predominant form in humans is CoQ10, which contains 10 isoprene units, whereas in mice, the major isoform is coenzyme Q9 (CoQ9), with nine isoprene units (2, 3). CoQ acts as both an electron acceptor from the tricarboxylic acid cycle and subsequently as an electron carrier on the inner mitochondrial membrane, transferring electrons from complex I (NADH:ubiquinone oxidoreductase) and complex II (succinate dehydrogenase) to complex III (ubiquinol-cytochrome *c* reductase) of the mitochondrial respiratory chain, which powers ATP synthesis. In its reduced form (ubiquinol), CoQ acts as a lipid-phase antioxidant, preventing the peroxidation of membrane lipids and regenerating vitamins E and C (4, 5, 6). In addition, CoQ receives electrons from enzymes involved in amino acid breakdown (PRODH), nucleoside synthesis (DHODH), choline metabolism (CHDH), flavoprotein dehydrogenase (ETFDH), glycolysis (G3PDH), and sulfate metabolism (SQOR), which all serve as pivotal roles in the Q junction (7).

Primary CoQ10 deficiency is a rare mitochondrial disorder caused by pathogenic variants in genes encoding proteins associated with the biosynthesis of CoQ10 or its regulation, including *COQ4* (8, 9). The disorder is both genetically and clinically heterogeneous and often presents as a multisystem disease with encephalopathy, cerebellar ataxia, myopathy, cardiopathy, and nephropathy as major presentations. Preliminary evidence suggests that high-dose oral CoQ10 supplementation could at best partially abrogate some disease manifestations (10, 11, 12) but does not prevent disease progression. It is postulated that oral CoQ10 therapy has limited clinical applicability because of the molecule's poor gastrointestinal absorption, low systemic bioavailability, and limited tissue uptake (13). From a mechanistic level, CoQ10 deficiency negatively affects electron transport chain function, sulfide oxidation, pyrimidine synthesis, and lipid metabolism. It also increases reactive oxygen species and oxidative stress and promotes mitochondrial turnover through mitophagy and activates mitochondrial unfolded protein response (14). This stress-induced adaptation reinforces the necessity of restoring CoQ into tissues and subcellular compartments to counteract chronic metabolic dysfunction and improve clinical outcomes.

BPM31510 is a lipid nanoparticle containing 4% oxidized CoQ10, dimyristoyl phosphatidylcholine, and poloxamer-188, forming nanoparticulate vesicles between 50 and 70 nm in size. It has been developed to overcome the poor pharmacokinetics and quality governance associated with conventional CoQ10 supplements, which are not pharmaceutically formulated, are not made under cGMP conditions, and do not concentrate in pharmacologically active levels in target tissue and appropriate organelles. In order to bioanalytically characterize CoQ10 and its metabolites and precursors, we have created a quinomics platform, specifically integrating targeted LC–MS/MS with MALDI-MS imaging (MSI) to assess quinone metabolism and localization. MALDI-MSI offers a label-free spatially resolved detection of small molecules, lipids, and metabolites from tissue sections in an analysis (15, 16, 17). In previous studies, MALDI-MSI has been used to probe spatial metabolic reprogramming in a variety of diseases and investigate regionally defined redox dynamics in mitochondrial disease models (18, 19, 20, 21).

Herein, we used our integrated platform to assess BPM31510 efficacy in increasing CoQ pool levels in a novel mouse model of primary CoQ deficiency because of a homozygous mutation in *Coq4* that leads to dysregulated CoQ biosynthesis across various tissues. The *Coq4* mouse model is currently being characterized phenotypically; thus, the goal of the current investigation was to assess tissue CoQ level deficiency and the level of CoQ restoration achieved across tissues. Using LC-MS/MS and MALDI-MSI, we assessed the systemic and spatial restoration of CoQ species post-BPM31510 treatment. Our analysis showed that BPM31510 can increase CoQ10 in the brain, especially the cerebellum, heart, and kidney. Furthermore, spatial quinomics demonstrated region-specific biodistribution and metabolic rescue by BPM31510, indicating that BPM31510 is a promising candidate for the treatment of primary CoQ10 deficiency and highlighting the potential for leveraging MSI to advance mitochondrial therapeutics. Taken together, these analyses demonstrated that spatial metabolomics allow for assessment of systemic tissue targeting and hence the potential for CoQ10 restoration therapy in primary CoQ10 deficiency.

## Materials and methods

### Animal studies and treatment

C57BL/6J *Coq4*^*F147C*^ knock-in mice were developed commercially by Ingenious Targeting Laboratories (Ronkonkoma, NY) using homologous recombination to introduce a c.440T>G (p.F147C) mutation into mouse embryonic stem cells. Targeted iTL BF1 (C57BL/6 FLP) embryonic stem cells were microinjected into Balb/c blastocysts. Resulting chimeras with a high percentage of black coat color were mated to C57BL/6 WT mice to generate germline Neo-deleted mice. Specific primer sets were used to screen mice for the deletion of the Neo cassette and for the absence of the FLP transgene. Tail DNA samples from positive mice were amplified to detect the presence of the introduced point mutation using LOX1 and RNEOGT primers. LOX1 is on the long homology arm upstream of the distal LoxP site. RNEOGT is downstream inside the remaining Neo cassette. Heterozygous mice were confirmed for germline Neo deletion and FLP absence (Supplemental Fig. S1).

**Fig. 1:**
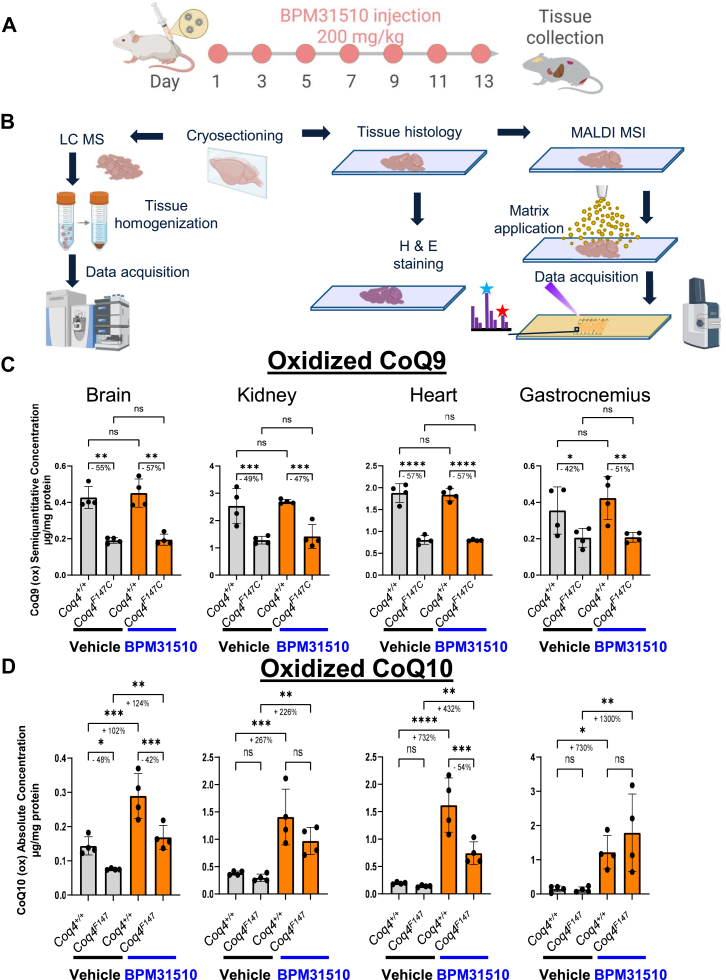
Experimental design and multimodal analysis pipeline to assess the efficacy of BPM31510 in increasing CoQ10 levels in *Coq4*^*+/+*^ or *Coq4*^*F147C*^ mice. A: *Coq4*^*F147C*^ mice received intraperitoneal (IP) doses of BPM31510 (200 mg/kg BPM31510) every other day for a total of 2 weeks. Tissues were collected at the end of the treatment (day 13). B: Tissue samples were prepared in parallel for LC-MS/MS, histology, and MALDI MSI. For the MALDI-MSI analysis, tissues were cryosectioned, matrix applied, and data acquired to visualize the spatial distributions of CoQ10 and related metabolites. Adjacent sections were utilized for H&E staining. For LC-MS/MS analysis, ten 10 μm tissue sections adjacent to the MSI plane were collected, homogenized, and extracted to quantify quinone species and assess the impact of BPM31510 treatment on tissue CoQ levels. The graphical abstract was designed using BioRender (www.BioRender.com). C: LC-MS/MS analysis of oxidized CoQ9 in brain, kidney, heart, and gastrocnemius from *Coq4*^*+/+*^ and *Coq4*^*F147C*^ mice treated with vehicle or BPM31510. D: LC-MS/MS quantification of oxidized CoQ10 in brain, kidney, heart, and gastrocnemius from *Coq4*^*+/+*^ and *Coq4*^*F147C*^ mice treated with vehicle or BPM31510. Data are represented as micrograms per milligram of protein (mean ± SD, n ≥ 4 per group). ∗*P* < 0.05, ∗∗*P* < 0.01, ∗∗∗*P* < 0.001; ns = not significant (one-way ANOVA). Ox, oxidized.

Mice were housed and bred according to international standard conditions, maintained with a 12-h light, 12-h dark cycle, and provided with food and water ad libitum. All experiments were performed according to a protocol (AC-AABZ2653) approved by the Columbia University Institutional Animal Care and Use Committee and were consistent with the National Institutes of Health Guide for the Care and Use of Laboratory Animals. Studies were performed in 1-month-old male and female *Coq4*^*F147C*^ mice along with wild-type littermates (*Coq4*^*+/+*^) (N ≥ 4 per group). BPM31510 was administered by intraperitoneal injection at a dose of 200 mg/kg once every 2 days for a total of 13 days (seven injections) (Fig. 1A). The intraperitoneal route of administration was chosen since it has been historically deemed comparable to intravenous infusions, which are given in humans, and has a greater ease of use in mice. Animals in the vehicle-treated group received matching injections of PBS. By day 13, animals were euthanized by CO_2_, followed by cervical dislocation, and a large number of tissues (brain, heart, kidney, and muscle) were collected, snap-frozen in liquid nitrogen, and stored at −80°C until analyzed. Skeletal muscles (gastrocnemius) were frozen in isopentane precooled to approximately −160°C using dry ice.

### LC-MS/MS analysis of quinone metabolites

Brain, kidney, heart, and gastrocnemius samples (100 μm each, cryosectioned) were homogenized using an Omni Bead Ruptor (Omni International) in 300 μl 0.1X PBS kept at 4°C, and lipids were extracted from 100 μl homogenates using 300 μl Optima-LCMS grade isopropanol and 50 μl Optima-LCMS grade methanol, delivering 0.01 μg of the isotopically labeled (d9) CoQ10 as the internal standard (ISTD, Sigma-Aldrich). Samples were subsequently vortexed and centrifuged, and the supernatants were isolated and stored as described above. UHPLC-MS/MS analyses were performed on an Agilent 1290 Infinity HPLC coupled to a Thermo Q-Exactive Plus Mass Spectrometer. A 30-min binary multistep gradient was used with A: 100% water with 5 mM ammonium formate and 0.1% formic acid and B: 90/10 isopropanol/acetonitrile with 10 mM ammonium formate and 0.1% formic acid. The column was a Waters Acquity HSS-T3, 100 mm × 2.1 mm × 1.8 μm. The mass spectrometer was operated in parallel-reaction monitoring mode at 17,500 resolution, positive ion with spray voltage +3.5 kV, quadrupole isolation masses 880.71821 (CoQ10(ox)), 812.6557 (CoQ9(ox)), and 889.77383 (ISTD) with an isolation window of ±1.5 Da for the [M + NH_4_]^+^ adducts of all three species, and collision energy of 30 eV. Quinones were extracted from tissues using isopropanol and deuterium-labeled CoQ10 as an ISTD.

Absolute quantification of oxidized CoQ10 was performed via isotope dilution MS. A 16-point standard curve was included in the analysis, ranging from 0.095 ng/ml up to 9.5 μg/ml oxidized CoQ10. Four injections of each level were included in the analysis, and a weighted linear regression was applied. Absolute (CoQ10(ox)) concentrations were normalized to total protein (expressed as micrograms of analyte per milligram of total protein) as determined by a total protein assay (Pierce BCA Kit; ThermoFisher).

### MALDI-MSI for quinone metabolites

Tissue samples were cryosectioned at a thickness of 10 μm sections using a cryostat (Cryostar Nx50 cryostat), and sections were thaw-mounted onto indium tin oxide-coated glass slides. For CoQ10 imaging, tissue was first coated with a deuterated CoQ10 (d9-CoQ10) at a concentration of 500 μM onto each tissue section before applying the matrix, 1,5-diaminonaphthalene, at a concentration of 10 mg/ml in 80:20 (v/v) acetonitrile:water. Both the d9-CoQ10 and matrix were uniformly applied using a Tissue Matrix Sprayer (HTX Technologies). The Tissue Matrix Sprayer for d9-CoQ10 was under the following parameters: nozzle temperature of 77°C, flow rate of 0.1 ml/min and 2 passes; and for the matrix: nozzle temperature of 77°C, flow rate of 0.1 ml/min, and 6 passes. After the application of both the standard and matrix, the slides were analyzed for imaging. MALDI-MSI was performed with a timsTOF fleX mass spectrometer (Bruker Daltonics) in negative ion mode with a spatial resolution of 20 μm. Data acquisition was optimized for laser frequency at 10,000 and laser shots at 600, and the spectra were acquired across the *m/z* range of 100 to 3,000. Laser energy and collision energy were also optimized to ensure maximum ion yield. CoQ species were annotated from comparisons of accurate mass, isotopic pattern, and MS/MS of standards.

### Data analysis

Raw imaging data were imported into SCiLS Lab (version 2025b Pro; Bruker Daltonics) for preprocessing, including baseline correction, normalization (to total ion count), peak picking, and spectral alignment. Ion images were generated for [oxidized CoQ10]^-^ (*m/z* of 862.6813), [oxidized CoQ9]^-^ (*m/z* of 794.6219), and [Heme B-H]^-^ (*m/z* of 615.1176) using negative mode data. Unsupervised segmentation was performed using the k-means clustering algorithm, which enables visualization and comparison of cluster-specific spectra and subsequent correlation with histological features.

### Histology and region of interest definitions

For H&E staining, serial tissue sections were prepared to visualize anatomical structures using anatomical markers to establish regions of interest (ROIs). ROIs were defined matching the landmarks previously established as known in terms of histological landmarks for each organ system, that is, cerebellar (white/gray matter, molecular layer, granular layer, fiber tracts, and vascular), renal functions (cortex/medulla), and myocardial (layers); and these ROIs were overlayed on the imaging data to enable spatially resolved semiquantification of CoQ signal intensity.

### Statistical analysis

Statistical comparisons of oxidized CoQ10 across treatment groups were conducted using one-way ANOVA in GraphPad Prism (GraphPad Software, LLC [Version 10.6.1]), with significance defined as *P* < 0.05. Graphs were created in GraphPad Prism.

## Results

### BPM31510 treatment increases tissue CoQ10 levels in *Coq4*^*F147C*^ mice

To evaluate the depletion of quinone pools and the degree to which BPM31510 increases CoQ10 in *Coq4*^*F147C*^ mice, LC-MS/MS was performed on tissue homogenates from the BPM31510 treatment study (Fig. 1A, B). Tissue levels of oxidized CoQ9, the primary endogenously synthesized quinone species in mice, were examined to determine the degree of native quinone depletion. Vehicle-treated *Coq4*^*F147C*^ mice were significantly depleted of oxidized CoQ9 levels compared with *Coq4*^*+/+*^ in the brain (−55%), kidney (−49%), heart (−57%), and gastrocnemius (−42%) (Fig. 1C). BPM31510 treatment did not significantly modify oxidized CoQ9 levels across any of the tissues examined. This lack of increase in CoQ9 values after BPM31510 treatment is consistent with the nature of BPM31510, which only contains CoQ10 and does not replenish CoQ9 directly.

*Coq4*^*F147C*^ mice had significantly lower amounts of oxidized CoQ10 levels when compared with the *Coq4*^*+/+*^ controls in brain (−48%) samples (Fig. 1D). In *Coq4*^*+/+*^, treatment with BPM31510 increased oxidized CoQ10 levels compared with the vehicle by 102% in the brain, 267% in the kidney, 732% in the heart, and 730% in the gastrocnemius (Fig. 1D). In *Coq4*^*F147C*^ mice, BPM31510 treatment raised oxidized CoQ10 levels compared with vehicle by 124% in brain, 226% in kidney, 432% in heart, and 1300% in gastrocnemius, increasing the overall CoQ pool to the levels that were either comparable (brain) to or higher (kidney, heart, and gastrocnemius) than those observed in *Coq4*^*+/+*^ control animals (Fig. 1D).

In summary, these results strongly demonstrate that BPM31510 preferentially increases oxidized CoQ10 levels in metabolically active tissues with a minimal effect on CoQ9 levels in the tissues measured. This pattern suggests specificity of intervention and effective systemic and tissue-level targeting of BPM31510, highlighting its potential to pharmacologically correct CoQ deficiency.

### BPM31510 treatment increases CoQ10 in multiple brain regions, including the cerebellum

**Fig. 2:**
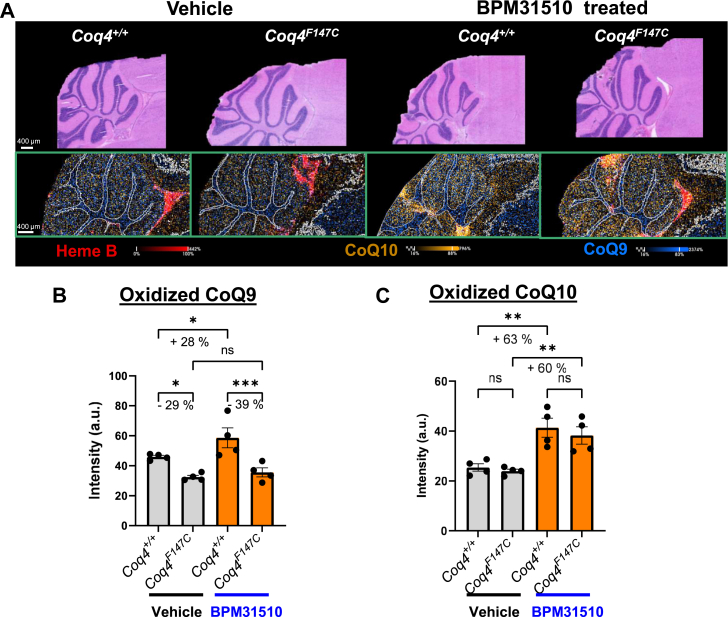
BPM31510 treatment increases CoQ10 levels in the cerebellum. A: Representative H&E sections and MALDI-MSI composite ion images of sagittal cerebellum sections from *Coq4*^*+/+*^ and *Coq4*^*F147C*^ mice treated with vehicle or BPM31510. Ion distributions of heme B (*red*), CoQ10 (*yellow*), and CoQ9 (*blue*) are shown. Average ion signal of (B) CoQ9 and (C) CoQ10 in the whole cerebellar region from *Coq4*^*+/+*^ and *Coq4*^*F147C*^ mice treated with vehicle or BPM31510. Data are represented as intensity (a.u.) (mean ± SD, n ≥ 4 per group). ∗*P* < 0.05, ∗∗*P* < 0.01, ∗∗∗*P* < 0.001; ns, not significant (one-way ANOVA).

Although LC-MS offers sensitive quantitation of CoQ species in homogenized tissue, it does not provide the spatial resolution and cannot separate functionally distinct brain regions because of the need for microdissection and sensitivity limitations. Thus, we further expanded our assessment using MALDI-MSI by utilizing ion segmentation to examine the anatomical features from the tissue sections and localize CoQ species. To gain deeper insight into regional CoQ distribution, we conducted a focused analysis of the cerebellum, a brain region frequently impacted in primary CoQ deficiency. Composite ion images of oxidized CoQ9, CoQ10, and heme B are shown in the cerebellum (Fig. 2A). Treatment with BPM31510 resulted in a 28% increase in CoQ9 levels in the *Coq4*^*+/+*^ mice, with no significant CoQ9 changes in *Coq4*^*F147C*^ mice being detected (Fig. 2B). In contrast, robust elevation of CoQ10 levels was observed in both *Coq4*^*+/+*^-treated mice (63% increase) and in *Coq4*^*F147C*^ mice (60% increase) as compared with vehicle control groups (Fig. 2C). The MSI findings are consistent with the bulk LC-MS/MS results, which showed a significant increase in CoQ10 in the *Coq4*^*+/+*^ and *Coq4*^*F147C*^ brain tissue after treatment. This degree of CoQ10 enrichment in the cerebellum is particularly noteworthy, as it reflects substantial accumulation of the therapeutic in a brain region critically affected in primary CoQ deficiency syndromes, particularly those presenting with ataxia. While LC-MS/MS provides a total amount of CoQ10 in a bulk tissue, MALDI-MSI provides a necessary spatial resolution, which indicates that BPM31510 crosses the blood-brain barrier and is distributed in the cerebellar tissue. This indicates that BPM31510 has a demonstrated ability to deliver CoQ10 to brain regions, such as the cerebellum, which is particularly vulnerable to low levels of CoQ and is often affected in primary CoQ deficiency, from the multisystemic presentations to the isolated ataxic phenotype.

**Fig. 3:**
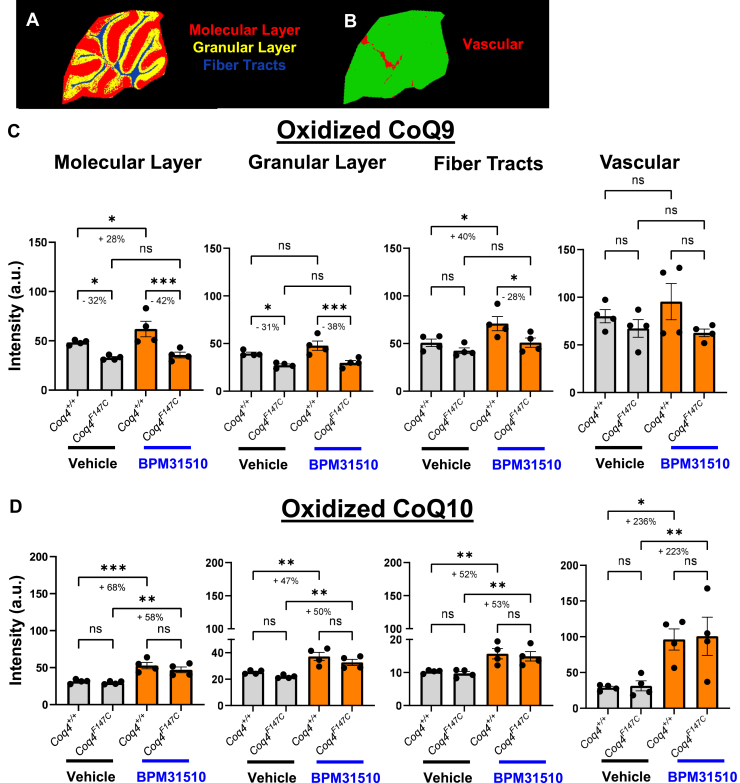
Spatial distribution and semiquantitative analysis of CoQ species within the cerebellum subregions. Segmentation map defining the molecular layer, granular layer, the fiber tracts (A), and vascular (B) regions that were utilized for quantifying spatial distribution. Semiquantitative MALDI-MSI intensity analysis of (C) CoQ9 and (D) CoQ10 in the defined cerebellar regions. Data are represented as intensity (a.u.) (mean ± SD, n ≥ 4 per group). ∗*P* < 0.05, ∗∗*P* < 0.01, ∗∗∗*P* < 0.001; ns, not significant (one-way ANOVA).

To identify the specific cerebellar substructures responsive to BPM31510 treatment, we further segmented the molecular layer, granular layer, the fiber tracts, and vascular regions of the cerebellum (Fig. 3A, B). Post-BPM31510 administration, we observed significant CoQ9 increases in the molecular layer (+28%) and fiber tracts (+40%) in *Coq4*^*+/+*^ mice compared with vehicle controls (Fig. 3C). *Coq4*^*F147C*^ mice did not exhibit any enrichment of the CoQ9 signal in any cerebellar compartment after treatment (Fig. 3C). Importantly, BPM31510 treatment yielded significant and consistent increases in oxidized CoQ10 in all cerebellar compartments in both *Coq4*^*+/+*^ and *Coq4*^*F147C*^ groups compared with respective vehicle controls (Fig. 3D). In the molecular layer, the oxidized CoQ10 signal intensity increased by 68% in *Coq4*^*+/+*^ and 58% in *Coq4*^*F147C*^ mice; in the granular layer, an increase of 47% in *Coq4*^*+/+*^ and 50% in *Coq4*^*F147C*^ mice was observed. Similarly, we detected higher CoQ10 accumulation in the fiber tracts of *Coq4*^*+/+*^ mice (+52%) and *Coq4*^*F147C*^ mice (+53%) versus control animals. Moreover, the vascular compartment exhibited the greatest elevation of oxidized CoQ10 signal, increasing by 236% in *Coq4*^*+/+*^ and 223% in *Coq4*^*F147C*^ animals (Fig. 3D). These data demonstrate the high cerebellar uptake of BPM31510, which results in the robust increase of CoQ10 levels in a region particularly vulnerable to CoQ deficiency.

In addition, we were able to delineate and capture oxidized CoQ9 and CoQ10 signal intensities in white matter, gray matter, and vascular components of the brain (Supplemental Figs. S2, S3A). The composite ion maps indicate that the oxidized CoQ10 signal in BPM31510-treated wild-type and mutant mice extends farther than the heme B regions, which serve as a vascular reference (Supplemental Fig. S2A), again, providing evidence of blood-brain barrier permeability and uptake into the brain parenchyma for BPM31510. Notably, this level of tissue segmentation and spatial assessment is not possible with bulk analysis by LC-MS. To further evaluate regional CoQ distribution, we analyzed average signal intensities of oxidized CoQ9 and CoQ10 in segmented white matter, gray matter, and vascular-rich areas of the brain (Supplemental Fig. S2C, D). As for oxidized CoQ9, treatment with BPM31510 had a significant effect in *Coq4*^*+/+*^ mice, increasing by 23% in white matter and 19% in gray matter regions. There was no significant change in the vascular regions in the wild-type group (Supplemental Fig. S2C). *Coq4*^*F147C*^ mice, however, did not show any significant alterations in CoQ9 signal post-treatment (Supplemental Fig. S2C). As for CoQ10, treatment with BPM31510 resulted in significantly elevated signal intensities in *Coq4*^*+/+*^ mice by 41%, 50%, and 263% in gray matter, white matter, and vascular-enriched regions, respectively. Likewise, in *Coq4*^*F147C*^ mice, CoQ10 levels increased by 33% in white matter and 48% in gray matter, and vascular enrichments did not show significant accumulation (Supplemental Fig. S2D).

Overall, ion segmentation allowed us to semiquantify CoQ species in specific regions of the brain tissue, demonstrating the ability of MALDI-MSI to discriminate and analyze spatial biodistribution of compounds. Consistent with the LC-MS/MS analysis, these data demonstrate that BPM31510 treatment effectively targets CoQ10 to the brain. While CoQ9 does not vary greatly following exogenous CoQ10 administration in *Coq4*^*F147C*^ animals, CoQ10 shows an increased signal in multiple anatomically defined brain areas, most significantly in gray and white matter of both genotypes.

### BPM31510 treatment increases CoQ10 levels spatially in kidney subregions

**Fig. 4:**
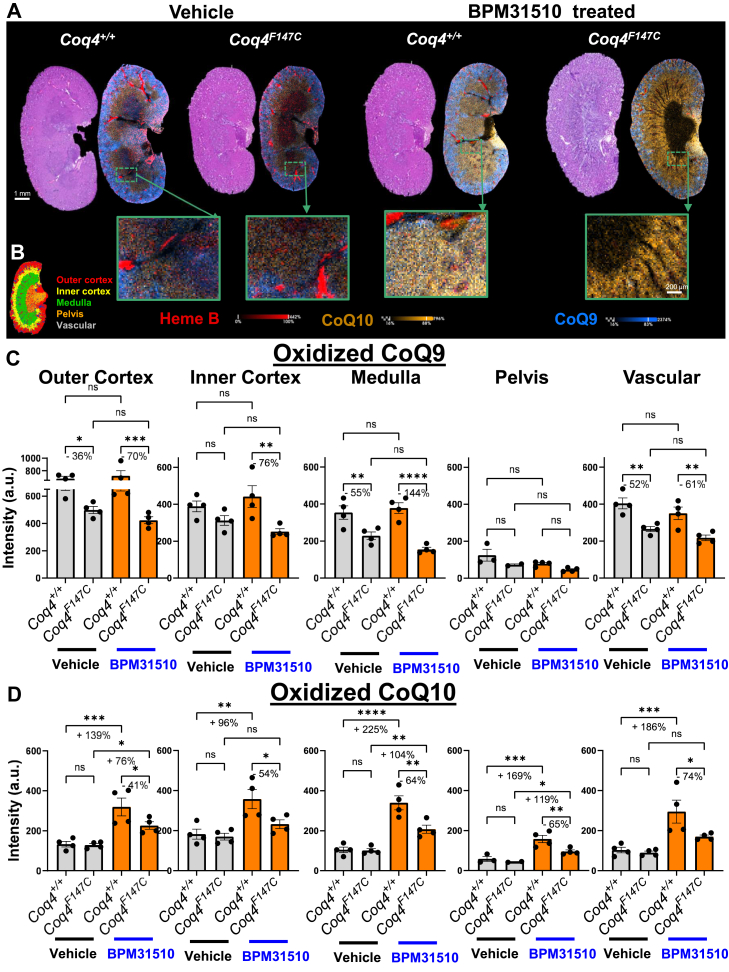
Spatial distribution and semiquantitative analysis of CoQ species within the kidney. A: Representative H&E sections and MALDI-MSI composite ion images of sagittal kidney sections from *Coq4*^*+/+*^ and *Coq4*^*F147C*^ mice treated with vehicle or BPM31510. Ion distributions of heme B (*red*), CoQ10 (*yellow*), and CoQ9 (*blue*) are shown. B: Segmentation map defining the outer cortex (*red*), inner cortex (*yellow*), medulla (*green*), pelvis (*orange*), and vascular (*white*) subregions for quantifying spatial distribution. Semiquantitative MALDI-MSI intensity analysis of (C) CoQ9 and (D) CoQ10 in the defined kidney regions. Data are represented as intensity (a.u.) (mean ± SD, n ≥ 4 per group). ∗*P* < 0.05, ∗∗*P* < 0.01, ∗∗∗*P* < 0.001; ns, not significant (one-way ANOVA).

LC-MS measured significant increases in oxidized CoQ10 levels in the whole kidney after BPM31510 treatment. MALDI-MSI ion maps of CoQ9, CoQ10, and heme B from kidney sections from each *Coq4*^*+/+*^ and *Coq4*^*F147C*^ mice exhibited distinct spatial regionalization within the kidney (Fig. 4A, Supplemental Fig. S3B). In general, ion tissue distribution suggests that the CoQ10 regions extend beyond heme B, the marker of blood vessels, into the renal parenchyma, clearly indicating that BPM31510 is entering the tissue. Moreover, ion segmentation allowed the generation of spatial maps of anatomical kidney compartments, such as the outer cortex, inner cortex, medulla, pelvis, and vascular regions, to further specifically quantify the CoQ9 and CoQ10 localization post BPM31510 treatment (Fig. 4B).

When semiquantifying CoQ9 levels across segmented kidney regions, no statistically significant changes were observed in any anatomical compartments of both mutant and wild-type mice following BPM31510 administration (Fig. 4C), consistent with LC-MS/MS results, although CoQ9 levels independent of treatment were lower in *Coq4*^*F147C*^ mice in specific regions. However, CoQ10 levels were significantly increased after BPM31510 supplementation across multiple subregions (Fig. 4D). In *Coq4*^*+/+*^ mice, BPM31510 treatment raised the levels of CoQ10 by 139% in the outer cortex, 96% in the inner cortex, 225% in the medulla, 169% in the pelvis, and 186% in the vascular region compared with nontreated control animals. *Coq4*^*F147C*^ mice showed increases of 76%, 104%, or 119% for the outer cortex, medulla, and pelvis, respectively, with no statistically significant elevation in the inner cortex or vascular region after BPM31510 treatment. Overall, these data support that BPM31510 treatment enables an increase and potential repotentiation of CoQ10 levels in all regions of the kidneys, which are often adversely affected by CoQ10 deficiency.

### BPM31510 treatment increases CoQ10 levels in cardiac tissue

**Fig. 5:**
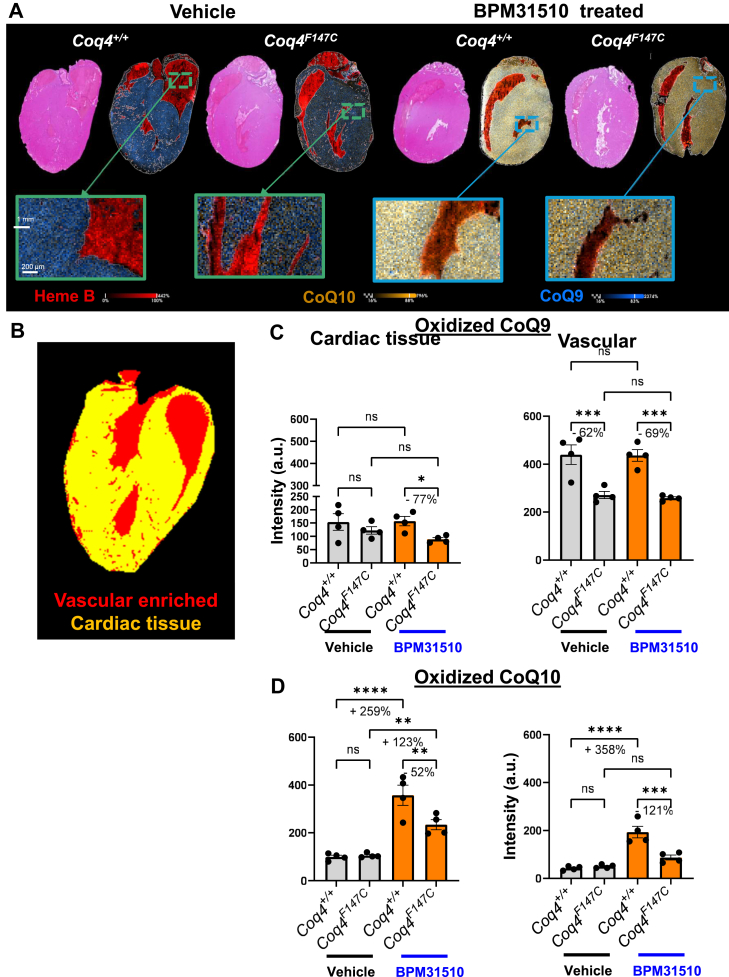
Spatial distribution and semiquantitative analysis of CoQ species within the heart. A: Representative H&E sections and MALDI-MSI composite ion images of cardiac cross-sections from *Coq4*^*+/+*^ and *Coq4*^*F147C*^ mice treated with vehicle or BPM31510. Ion distributions of heme B (*red*), CoQ10 (yellow), and CoQ9 (*blue)* are shown. B: Segmentation map defining the vascular-enriched (*red*) and myocardial (*yellow*) subregions for quantifying spatial distribution. Semiquantitative MALDI-MSI intensity analysis of (C) CoQ9 and (D) CoQ10 in the defined heart regions. Data are represented as intensity (a.u.) (mean ± SD, n ≥ 4 per group). ∗*P* < 0.05, ∗∗*P* < 0.01, ∗∗∗*P* < 0.001, ∗∗∗∗*P* < 0.0001; ns, not significant (one-way ANOVA).

To assess the spatial distribution of CoQ species within the heart, we applied MALDI-MSI to visualize and semiquantify CoQ9 and CoQ10 in myocardial and vascular regions in *Coq4*^*+/+*^ and *Coq4*^*F147C*^ mice post BPM31510 treatment as compared with vehicle controls (Fig. 5A, Supplemental Fig. S3C). Again, spatial maps demonstrate that the CoQ10 signal extended beyond the vascular regions delineated by heme B, indicating that CoQ10 successfully reached not only the blood vessels but also the surrounding myocardial tissue. Using MALDI-MSI ion segmentation, we could effectively separate cardiac and vascular tissue regions (Fig. 5B).

Assessment of CoQ9 levels demonstrated no significant changes post-BPM31510 treatment in both wild-type and mutant mice, similar to the kidney results (Fig. 5C). Conversely, CoQ10 levels in *Coq4*^*+/+*^-treated hearts showed a significant increase, with a 259% in the cardiac tissue and a 358% elevation in the vascular region. *Coq4*^*F147C*^-treated hearts showed a 123% enrichment in cardiac CoQ10 levels, whereas vascular CoQ10 levels remained unchanged in measured samples (Fig. 5D).

Overall, these data demonstrate that BPM31510 administration promotes targeted concentration of CoQ10 into the myocardial tissue. This is highly relevant, as this tissue is often adversely affected in primary CoQ10 deficiency, specifically in patients carrying mutations in the *COQ4* gene (22).

## Discussion

Primary CoQ10 deficiency is a genetically and clinically heterogeneous disease that frequently goes undiagnosed and is certainly underdiagnosed. In the last few years, the availability of genetic screening has enabled molecular diagnosis in a growing number of patients with this syndrome, unveiling new phenotypes and identifying defects in CoQ10 biosynthetic pathway genes not previously associated with human disease (23). Early diagnosis can enable rapid therapeutic intervention. In the current treatment of primary CoQ10 deficiency, high doses of CoQ10 supplementation are recommended. However, the therapeutic efficacy of oral CoQ10, particularly in the multisystemic infantile and neurological forms, is hampered by its poor bioavailability and limited organ uptake (24, 25, 26). Presently, several liposomal formulations have been developed but solely for the purpose of oral delivery, which has been hindered because of poor absorption. BPM31510 is the only formulation that has been in clinical development for intravenous administration to deliver significant amounts of CoQ10 within tissues in a pharmaceutical-grade formulation with enhanced stability.

Here, we simultaneously quantified and visualized CoQ species in high anatomical resolution in various tissues using targeted LC-MS/MS and spatial MALDI-MSI and evaluated the biodistribution and therapeutic potential of BPM31510 as a lipid nanoparticle containing oxidized CoQ10 in a novel mouse model of primary CoQ deficiency because of the *COQ4* mutation. COQ4 is a CoQ biosynthetic protein whose function is not completely understood. It has been shown that COQ4 displays oxidative decarboxylation activity at the first carbon of the quinone ring (27); however, its yeast homolog, Coq4, interacts with other Coq polypeptides and appears to be involved in organizing a high molecular mass complex of these polypeptides, required for CoQ biosynthesis in yeast (28, 29). Mutations in *COQ4* have emerged as a common cause of encephalocardiomyopathy, or cerebellar ataxia, conditions that are poorly responsive to CoQ supplementation (22, 30, 31, 32, 33). Cerebellar ataxia is not only a common manifestation of primary CoQ10 deficiency, associated with defects in PDSS2, COQ4, COQ5, COQ6, and COQ8A (34); low levels of CoQ10 have been reported also in cerebellar syndromes caused by mutations in genes not related to CoQ10 synthesis (secondary CoQ10 deficiencies), including ataxia oculomotor apraxia 1 (35, 36, 37), multiple system atrophy (38, 39), and GEMIN5-related disease (40). Therefore, the cerebellum is particularly vulnerable to CoQ10 deficiency. However, besides one report of the regional distribution of CoQ levels in the rat brain showing lower CoQ content in the cerebellum compared with other areas (41), a correlation between tissue concentration and phenotype leading to selective damage of this organ remains unclear. COQ8A-related cerebellar ataxia is the most common primary CoQ10 deficiency; however, the levels of CoQ in the cerebellum of Coq8a knock-out mice are not altered (42). We have generated and started to characterize a Coq4 knock-in mouse carrying the p.F147C mutation, whose homolog human mutation causes encephalopathy and cerebellar ataxia (43). Our studies show profound widespread CoQ depletion in 1-month-old Coq4 mutant mice compared with controls, indicating the potential use of this novel mouse model for investigating the mechanisms responsible for specific tissues' vulnerability to CoQ deficiency and for therapeutic testing.

We simultaneously quantified and visualized CoQ species in various tissues using targeted LC-MS/MS and spatial MALDI-MSI. The incorporation of MALDI-MSI allows proof of principle to investigate the distribution of endogenous CoQ, thus helping to evaluate the selective cell and tissue vulnerability to decreased levels of CoQ and the spatial dynamics of CoQ10 uptake and biodistribution of a putative therapeutic delivery in a more cohort-driven manner. Conventional strategies to assess drug exposure are bulk measurements that ignore regional compartmentalization. Our spatially resolved measure demonstrated differences in CoQ10 distribution and uptake within a single organ and emphasized the need to link regional spatial context to mitochondrial dysfunction and the importance of pharmacodynamics in rare metabolic diseases.

We demonstrated that BPM31510 increased CoQ10 in the brain, heart, and kidney, all of which are impacted in patients with primary CoQ10 deficiency. Importantly, we observed an increase in CoQ10 levels in the cerebral white and gray matter, molecular and granular layers of the cerebellum, cardiac tissue, and renal cortex and medulla, making our studies relevant from a clinical point of view, as cerebellar ataxia, cardiomyopathy, and nephrotic syndrome are common manifestations of primary CoQ10 deficiency. Thus, we propose that the BPM31510 construct may have countered some of the low oral bioavailability and incomplete tissue targeting challenges associated with oral delivery of CoQ10. Although it has been reported that dietary supplementation with CoQ10 increases both CoQ9 and CoQ10 levels in mice, suggesting that these animals can demethylate the isoprenoid side chain of CoQ10 (44, 45), our results are consistent with previous studies showing that CoQ10 supplementation does not increase the levels of CoQ9 in mice (25, 26). These discrepancies might be due to differences in protocols of supplementation and/or the purity of the CoQ10 preparation. Further studies aimed at assessing mitochondrial dysfunction, including oxidative phosphorylation activity defects, in *Coq4*^*F147C*^ mice, and the efficacy of BPM31510 in rescuing studies is ongoing.

The current article does acknowledge several limitations of the study. First, the experimental model was utilized. At the present time, the *Coq4*^*F147C*^ mouse has not been fully characterized. Although a deficiency of CoQ was detected across various tissues, its detrimental effects at the level of the electron transport chain or other pathways associated with the CoQ junction were not evaluated. Therefore, we did not study the therapeutic effects of BPM31510 on mitochondrial function and “clinical” phenotype. Second, we did not compare the BPM31510 administration with oral CoQ10 delivery. This was due to the established literature demonstrating the inability of oral CoQ10 formulations to increase CoQ10 in the brain and kidney as well as to the number of oral formulations available. Thus, our main focus was to assess CoQ10 delivery via BPM31510 to tissues, such as the brain (including cerebellum), kidney, heart, and muscle, all variably affected in primary CoQ10 deficiency.

In summary, this work provides evidence to support BPM31510 as a potentially potent next-generation CoQ10 therapeutic that can overcome the significant limitations of nutraceutical oral treatment and provide systemic exposure to tissues in an effective pharmaceutical construct for patients with genetically characterized primary CoQ10 deficiencies. Importantly, this work also provides a preliminary framework for employing the combined approaches of spatial quinomics with quantitative MS for drug evaluation in preclinical models of complex, multisystem diseases, such as mitochondrial-based diseases. Further work remains in the development of MALDI-MSI-based methods, which can simultaneously measure reduced and oxidized CoQs, as well as comprehensive MS-based analysis of other quinone analytes (e.g., shorter prenyl chains and adjacent cellular mechanisms), which can provide further insights into CoQ metabolism. Our studies might be relevant for other more common related genetic disorders leading to mitochondrial dysfunction, in which secondary CoQ deficiency and mitochondrial dysfunction have been reported to contribute to the disease (46).

## Data Availability

MS data may be provided by the corresponding author upon reasonable request.

## Supplemental Data

This article contains supplemental data.

## Conflict of Interest

The authors declare that they have no conflicts of interest with the contents of this article.
