# Supplementary material for: Spatial mapping of CoQ10 repletion by BPM31510 in a genetic mouse model (Coq4F147C) of coenzyme Q deficiency
Source: J Lipid Res. 2026 Jan 29;67(3):100987. doi: 10.1016/j.jlr.2026.100987 (PMC13080569; doi:10.1016/j.jlr.2026.100987)
Supplement: Supplemental Figure S1 [file mmc1.pdf]

A

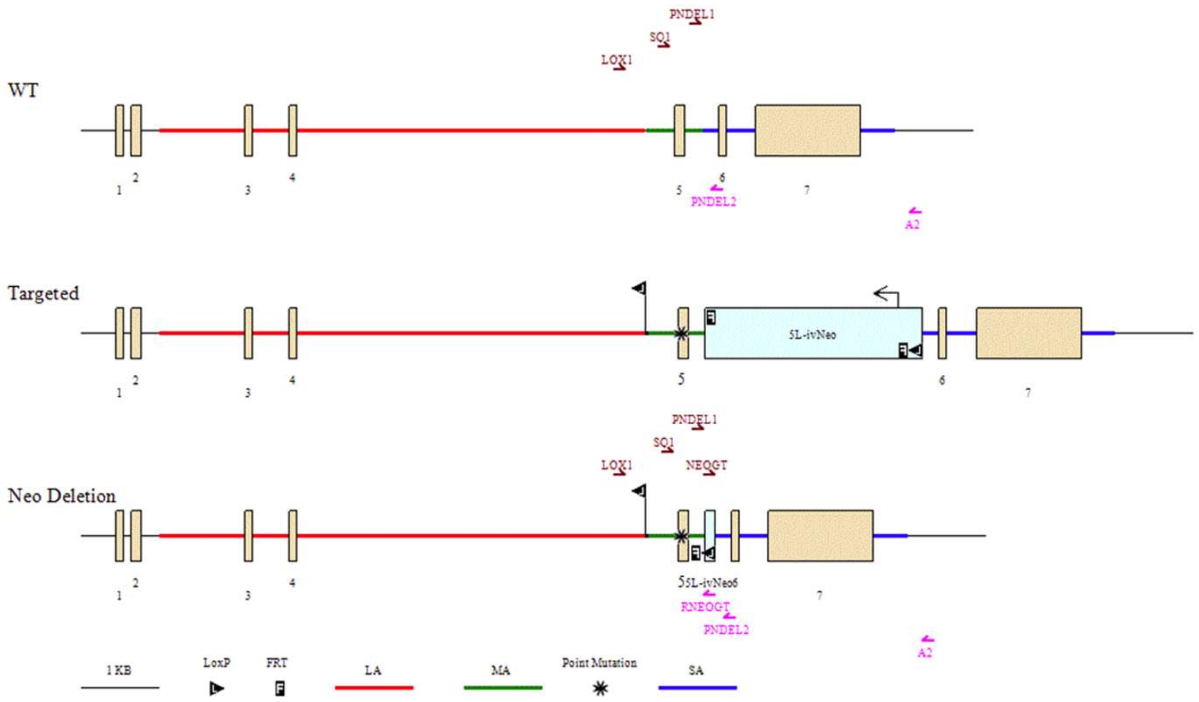

B

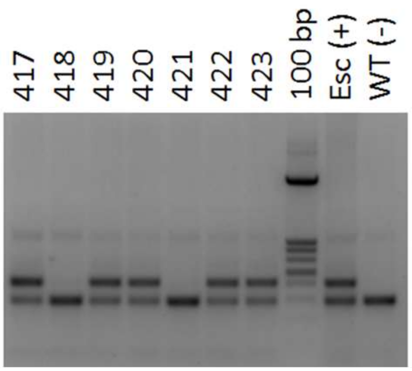

PNDEL1 / PNDEL2 (293 bp / 415bp)

C

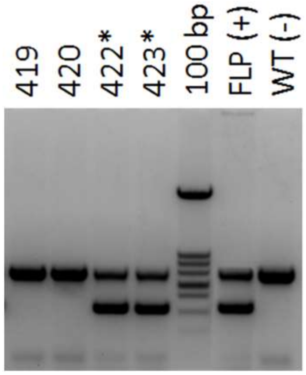

newFLP1 / newFLP2
(330 bp if FLP transgene present)
(*Asterisked mice are FLP present)

D

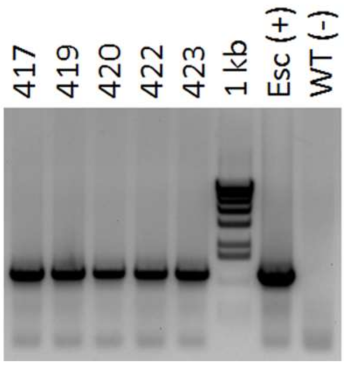

LOX1/ RNEOGT (1.12 KB)

E

```
Query  17     GGCAGGATTCTCNGTTAGGGTATGTGCGCAGTGGGAACATAGCTCAACGTCAACTAGCGG  76
              |||||||||||| |||||||||||||||||||||||||||||||||||||||||||||||
Sbjct  20663  GGCAGGATTCTCAGTTAGGGTATGTGCGCAGTGGGAACATAGCTCAACGTCAACTAGCGG  20722

Query  77     TGGGCCAGCAAGGCCCTGGGGGCTGATGGGAAGGAGTCCAAGGAGGGATTATGTTCTCCA  136
              ||||||||||||||||||||||||||||||||||||||||||||||||||||||||||||
Sbjct  20723  TGGGCCAGCAAGGCCCTGGGGGCTGATGGGAAGGAGTCCAAGGAGGGATTATGTTCTCCA  20782

Query  137    GAAGGTCTCTCCAGACACACGAGCACCTACACGCTGTGTGGACGATGAAGAGCTAGCCTA  196
              ||||||||||||||||||||||||||||||||||| ||||||||||||||||||||||||
Sbjct  20783  GAAGGTCTCTCCAGACACACGAGCACCTACACGCTTTGTGGACGATGAAGAGCTAGCCTA  20842

Query  197    TGTGATCCAGAGGTACCGTGAAGTGCACGACATGCTCCACACCCTGCTGGGCATGCCCAC  256
              ||||||||||||||||||||||||||||||||||||||||||||||||||||||||||||
Sbjct  20843  TGTGATCCAGAGGTACCGTGAAGTGCACGACATGCTCCACACCCTGCTGGGCATGCCCAC  20902

Query  257    CAACATGCTCGGTGAGTGCCGGCGGCCGGACAGGAGCCGGCAGGGCacccccaaccccac  316
              ||||||||||||||||||||||||||||||||||||||||||||||||||||||||||||
Sbjct  20903  CAACATGCTCGGTGAGTGCCGGCGGCCGGACAGGAGCCGGCAGGGCACCCCCAACCCCAC  20962

Query  317    cccacccccaGGTCTGTCTGAGAAGGCAGAATCCCAGTTCGTGCTTCTCTTTATCAGCTA  376
              ||||||||||||||||||||||||||||||||||||||||||||||||||||||||||||
Sbjct  20963  CCCACCCCCAGGTCTGTCTGAGAAGGCAGAATCCCAGTTCGTGCTTCTCTTTATCAGCTA  21022

Query: Sequencing data from PCR products
Sbjct: Respective targeted allele sequence
```
