# Supplementary figures and images for: Spatial mapping of CoQ10 repletion by BPM31510 in a genetic mouse model (Coq4F147C) of coenzyme Q deficiency
Source: J Lipid Res. 2026 Jan 29;67(3):100987. doi: 10.1016/j.jlr.2026.100987 (PMC13080569; doi:10.1016/j.jlr.2026.100987)

A

Not treated

BPM31510 treated

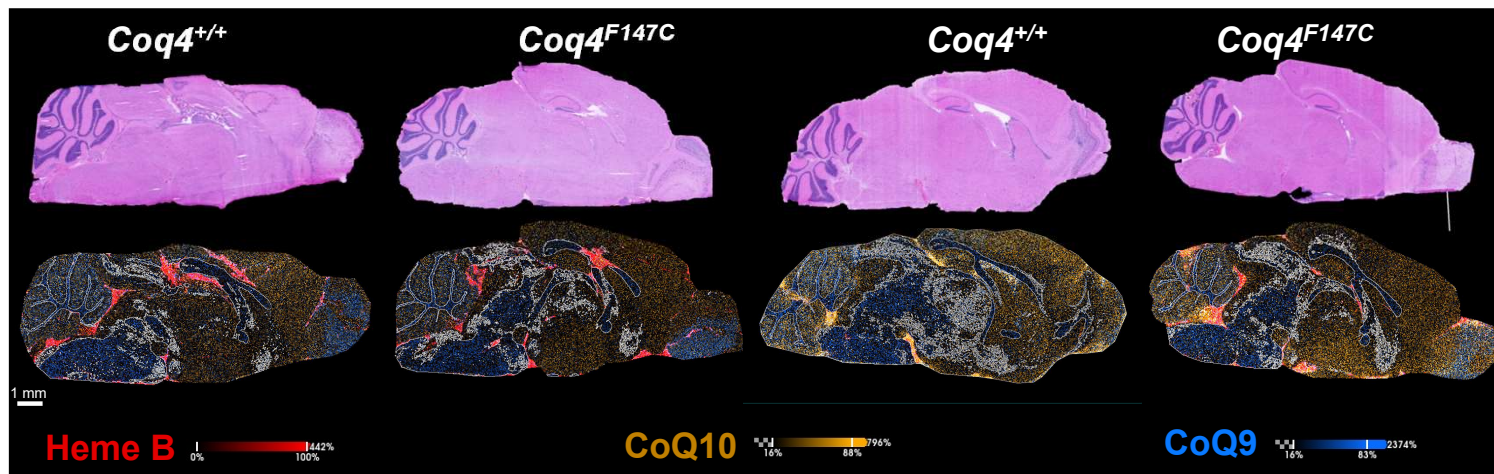

B

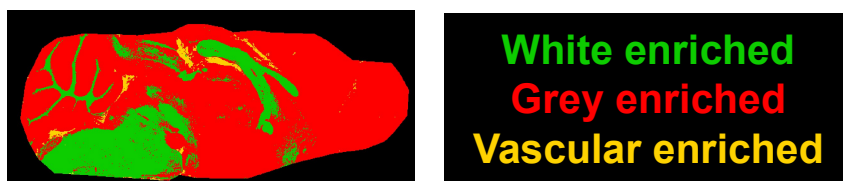

C

White enriched

Grey enriched

Vascular

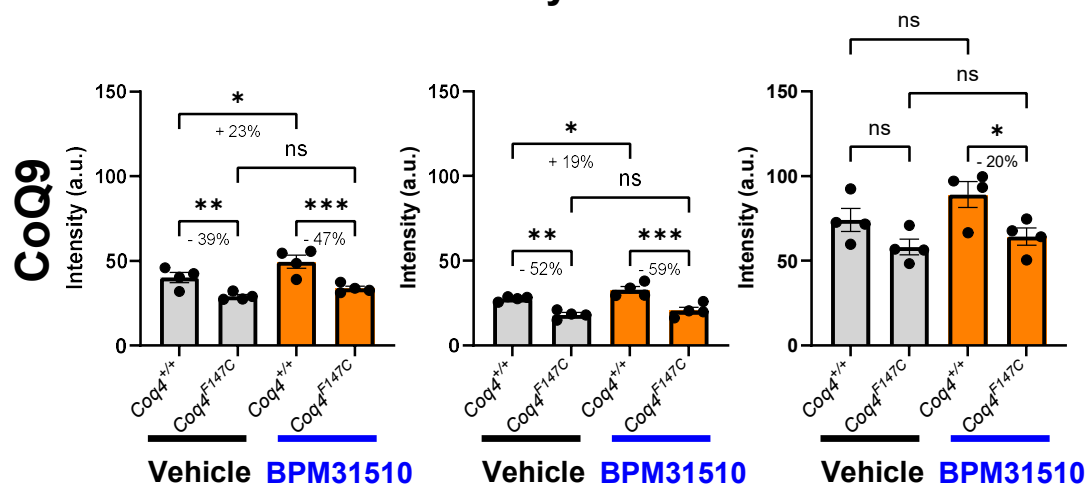

D

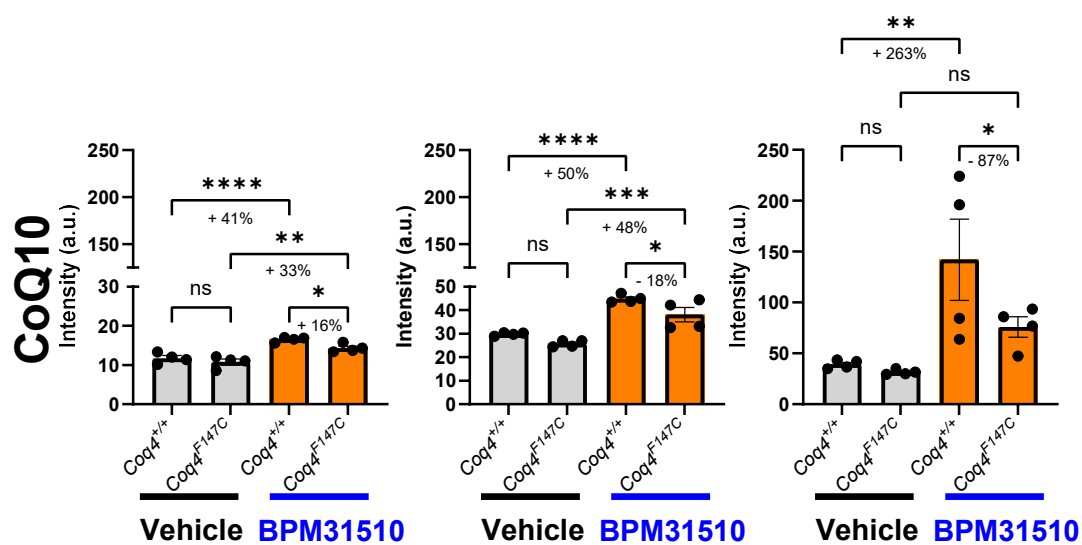

Supplement: Supplemental Figure S2 [file mmc2.pdf]

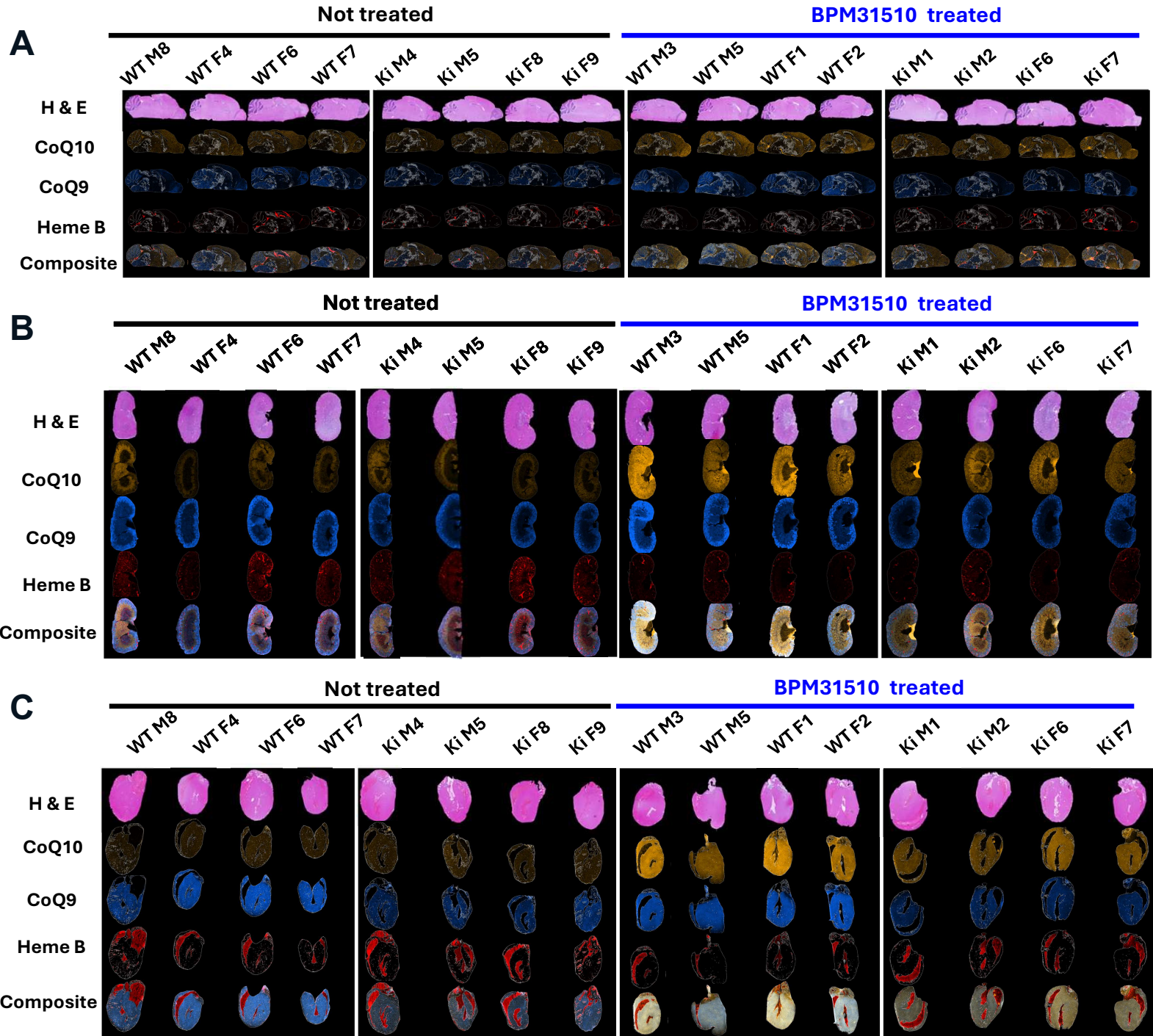

Supplement: Supplemental Figure S3 [file mmc3.pdf]
